# Effects of COVID-19 on non-communicable diseases and their surveillance in 10 African Union member countries

**DOI:** 10.4102/jphia.v16i1.861

**Published:** 2025-05-22

**Authors:** Adelard Kakunze, Fabian Moser, Betty K. Ingabire, Dumsani N. Mamba, Giselle Sarganas, Eva P. Renggli, Michael Zobi, Angela Fehr, Mohammed Abdulaziz

**Affiliations:** 1 Division of Disease Control and Prevention, Unit of Non-Communicable Diseases and Mental Health, Africa Centres for Disease Control and Prevention, Addis Ababa, Ethiopia; 2 Centre for International Health Protection, Robert Koch Institute, Berlin, Germany; 3 Department of Epidemiology and Health Monitoring, Unit of Physical Health, Robert Koch Institute, Berlin, Germany

**Keywords:** emergency preparedness and response, pandemic, mental health, NCDs, data

## Abstract

**Background:**

During health emergencies, continuity of care for non-communicable diseases (NCDs) and mental health (MH), informed by robust surveillance, is required. The COVID-19 pandemic revealed that many countries were ill-prepared in this respect.

**Aim:**

This study assesses the effect of COVID-19 on the continuity of care and surveillance of NCDs and MH in 10 African Union (AU) member states (MS) from the onset of the COVID-19 pandemic.

**Setting:**

The study was conducted in 10 AU MS, with two MS from each AU region.

**Methods:**

An online cross-sectional survey was developed. Member states were selected using stratified random sampling, and individual participants were selected from the ministries of health as national NCD and MH focal persons. Responses were analysed using descriptive statistics and thematic analysis.

**Results:**

All 10 MS responded. In two-thirds and half of participating MS, routine surveillance for NCDs and MH took place, respectively. During the COVID-19 pandemic, where data were available, several MS observed increases in NCD and MH risk factors and NCD mortality and MH morbidity. Half of the MS integrated NCDs and MH into emergency preparedness and response plans and activities.

**Conclusion:**

The MS had varied levels of national NCD and MH surveillance practices. Where data were available, most observed negative effects on NCDs and MH during the COVID-19 pandemic. Though the integration of NCDs and MH in emergency preparedness and response planning was limited, some countries responded with innovative measures to ensure continuity of care.

**Contribution:**

The study provides insights for improving public health surveillance and emergency response systems.

## Background

Non-communicable diseases (NCDs) and mental health (MH) conditions are significant public health threats on the African continent, causing substantive disease and mortality burdens.^1^ Forecasts anticipate the NCDs burden in Africa to increase further and to account for 35.9% of all years of life lost (YLL) across sub-Saharan Africa and 71.6% across North Africa and the Middle East by 2040.^1^

The COVID-19 pandemic exacerbated this situation, as several NCDs increase the risk of COVID-19 infection^2^ and severe courses of illness.^3^ To illustrate, across 13 African countries, the World Health Organization (WHO) found an average case fatality rate of 2.5% for COVID-19 patients, but a specific case fatality rate of 10% for COVID-19 patients who also had diabetes.^4^ The pandemic also impacted MH on the continent; there was an estimated increase of 23% of major depressive disorder cases across sub-Saharan Africa and 37% across North Africa and the Middle East during the pandemic compared to pre-pandemic baselines.^5^ Disruptions affected routine health services, access to medicines, screening and surveillance as countries partly reallocated healthcare staff and funding to the pandemic response, leaving other patients un- or underattended.^6,7^ Still, nearly 60% of countries in the World Health Organization, Africa and Eastern Mediterranean regions reported that continuity of NCD services was included in the list of essential health services during the pandemic.^6^ More than 90% of MS reported the inclusion of mental health and psychosocial support (MHPSS).^7^ Yet, only a minority of these countries implemented or evaluated their MHPSS strategy.^8^

The pandemic underscored the double burden of infectious and non-infectious diseases and the need for integrated detection and response.^9^ Non-communicable diseases and MH have to be included in emergency preparedness and response (EPR) planning to ensure continuity of care.^2,10,11,12,13,14^ Strengthening routine NCDs and MH surveillance is a prerequisite to determining relevant needs.^13,14,15^ The African Centres for Disease Control and Prevention (CDC) Strategy on NCDs, Injuries and Mental Health highlights the importance of both aspects and underlines the need for continental coordination, cooperation and investments in line with the New Public Health Order for Africa.^13^

Yet, data are scarce on the effects of COVID-19 on the continuity of care for NCDs and MH, on the integration of NCD and MH into EPR planning and on the foundational NCD and MH surveillance capacities and practices across the African continent. To start closing these data gaps, this study, based on an online survey with NCD and MH experts in 10 African Union (AU) MS, aimed to describe in these 10 countries: (1) routine national NCD and MH surveillance activities and data availability, (2) registered effects of the COVID-19 pandemic on major NCDs and MH conditions, risk factors, health services and surveillance activities from the onset of the COVID-19 pandemic to October 2022 and (3) the integration, if any, of NCDs and MH into countries’ national health EPR planning. The study further sought to identify and explore challenges and opportunities in strengthening NCD and MH surveillance, continuity of care and EPR integration.

## Research methods and design

### Study design and setting

A cross-sectional online survey with closed and open-ended questions was rolled out to national NCD and MH focal persons in 10 AU MS.

### Study population and sampling strategy

Selection criteria for countries were: (1) two countries from each AU region and (2) a balanced sample of anglophone as well as francophone countries. The country selection was carried out in a three-step process. Firstly, we allocated all AU countries to their respective AU region. Secondly, from each of the five regions, we randomly selected one country, irrespective of its first official language. Thirdly, for the Southern region, we randomly selected a second country, because this region has no francophone countries. For the Northern, Eastern, Western and Central regions, which have anglophone, francophone or bilingual countries, we formed subgroups consisting only of those countries that did not have the same main language as the country already selected in the first round. From these subgroups of the four remaining regions, we again selected one country per region. As a result, the following countries were selected: the Republic of South Africa, Republic of Botswana (Southern AU region); Democratic Republic of the Congo, Republic of Cameroon (Central); Kingdom of Morocco, Arab Republic of Egypt (Northern); Federal Republic of Nigeria, Republic of Senegal (Western); Seychelles and Republic of Rwanda (Eastern). Ministries of health from these countries were contacted. Individual participants were selected based on their technical area of expertise and their designation as national technical focal persons for NCDs as well as MH at the health ministries. They were requested to participate in the online survey between 10 October 2022 and 04 November 2022 and to elicit information from additional ministry technical officers as needed.

### Data collection

The survey was developed considering existing evidence, cross-country survey instruments^16,17^ and the strategic objectives of the Africa CDC NCD Injuries and MH Strategy.^13^ The questionnaire was reviewed and approved by the Africa CDC Advisory Committee on NCDs, injuries and MH (Advisory Committee NCD Injuries and MH). It comprised a total of 27 closed questions and 13 open-ended questions, which were organised into four subject-related blocks: (1) NCDs (governance, surveillance and COVID-19 impacts), (2) MH (governance, surveillance and COVID-19 impacts), (3) long/post COVID and (4) pandemic response, good practices, perspectives and support from Africa CDC. Within these blocks, the survey addressed the following questions: *Which NCDs and MH policies and/or strategies exist in MS? Which NCDs and/or MH data are available and where are gaps? Which data are available on the effects of COVID-19 on NCDs and MH?* This included sub-questions asking respondents to indicate whether the prevalence and incidence of major NCDs and MH conditions and their risk factors had increased, decreased or remained unchanged during the COVID-19 pandemic or whether these data were not available in their countries, using multiple-choice questions (no mandatory requirement to specify disease and risk factor frequencies in numbers or data sources, to increase feasibility). What action points have or shall be developed from these data? Are NCDs and/or MH part of national health EPR? A fifth block asked for feedback on the survey’s concept and its implementation, which shall be considered before potentially scaling up this instrument to additional AU countries. Filter questions guided national NCDs and MH focal persons to fill in the NCDs, respective to the MH block. All other blocks were filled by both groups. The questionnaire was developed in English and translated into French by bilingual Africa CDC staff.

### Data analysis

To benefit from all responses received, partially completed questionnaires were included in analyses. Responses to closed questions were analysed with descriptive statistics, calculating frequency distributions with a valid number of responses for each closed questionnaire item. Thematic analysis was used for full-text answers to open-ended questions. After removing MS identifying information, these answers were coded first for each MS and then compared across MS to identify and synthesise common themes. We excluded inconsistent responses from participants of the same MS (e.g., one suggests increased prevalence of anxiety during the COVID-19 pandemic, whereas another suggests a decrease) but included complementary responses (e.g., challenges in national NCD surveillance) in the analysis.

### Ethical considerations

Ethical clearance to conduct this study was obtained from the Africa Centres for Disease Control and Prevention (CDC) and AU member states (MS). Informed consent was obtained from all survey participants. Survey results were aggregated or anonymised so as to not reveal the identity of survey respondents. Prior approval for the survey was obtained from the Africa CDC science office.

## Results

All invited MS responded to the survey (*n* = 10/10). One MS did not respond to the NCD section. The survey received 32 responses; in some countries, more than one NCD or MH focal point filled in the questionnaire. Eighteen participants responded in their capacity as NCDs and 14 as MH focal points.

### Which non-communicable diseases and mental health policies and/or strategies exist?

Nearly all participating countries (*n* = 8/10) have NCD strategies, compared with 4 out of 10 MS having MH strategies (Table 1). Apart from one MS, which had no plan to develop a national NCD strategy, the remaining MS without an NCD (*n* = 1/10) or MH (*n* = 6/10) strategy plan on developing these national strategies.

**TABLE 1:** Availability of non-communicable disease and mental health strategies and cooperation of ministries and/or national public health institutes with external partners across member states countries.

Country	NCDs policies and/or strategies available (Yes or No)	MH policies and/or strategies available (Yes or No)	Ministry and/or national public health institute cooperates with external partners on NCD (Yes or No)	Ministry and/or national public health institute cooperates with external partners on MH (Yes or No)
A	Yes	No	Yes	Yes
B	Yes	No	No	Yes
C	Yes	Yes	Yes	Yes
D	Yes	Yes	Yes	Yes
E	Yes	No	Yes	No
F	No	No	Yes	No
G	Yes	Yes	Yes	Yes
H	Yes	No	No	-
I	Yes	No	Yes	Yes
J	-	-	Yes	Yes

NCDs, non-communicable diseases; MH, mental health; MS, member states.

Non-communicable diseases and MH offices or focal points usually coordinate with each other, and in half of MS, they also implement joint activities. Nearly all MS cooperate with external partners on NCDs (*n* = 8/10) and MH (*n* = 8/10) prevention and control, mostly with in-country universities and research institutes.

### Which non-communicable disease and mental health data are available and where are gaps?

The landscapes for NCDs and MH data are uneven across MS. Two-thirds of MS reported national surveillance activities for NCDs and a half for MH (Table 2). Where no national surveillance activities currently exist, countries usually discuss their development. Some MS use decentralised surveillance systems (e.g. DHIS-2 [District Health Information Software–2]) to track NCDs and MH from the community to the national level. Data analyses mainly occur at the ministry divisions or units for statistics or health information in collaboration with the units or programmes for NCDs and MH. Some MS work with external research or global multilateral organisations to analyse NCDs and MH data. Most MS produce health reports, some generate policy briefs and/or maintain dashboards.

**TABLE 2:** Availability of national non-communicable disease and mental health surveillance activities.

Country	Reports surveillance activities for NCDs (Yes/No)	Reports surveillance activities for MH (Yes/No)
A	No	Yes
B	No	No
C	Yes	Yes
D	Yes	Yes
E	Yes	No
F	No	No
G	Yes	Yes
H	Yes	No
I	Yes	No
J	-	Yes

NCDs, non-communicable diseases; MH, mental health.

Hospital records are the most frequent data source for NCDs and MH followed by population health surveys, including STEPwise surveys. The latest round of population health surveys was on average 7 years ago for NCDs and 13 years ago for MH. One-third of MS maintain cancer registries (Table 3).

**TABLE 3:** Availability of population health surveys for non-communicable diseases and/or mental health and of cancer registries across member states.

Country	Has conducted a population health survey (Yes or No)	Has cancer registry (Yes or No)
A	Yes	-
B	Yes	-
C	Yes	Yes
D	Yes	-
E	Yes	-
F	-	-
G	Yes	-
H	Yes	Yes
I	Yes	-
J	Yes	-

**FIGURE 1:**
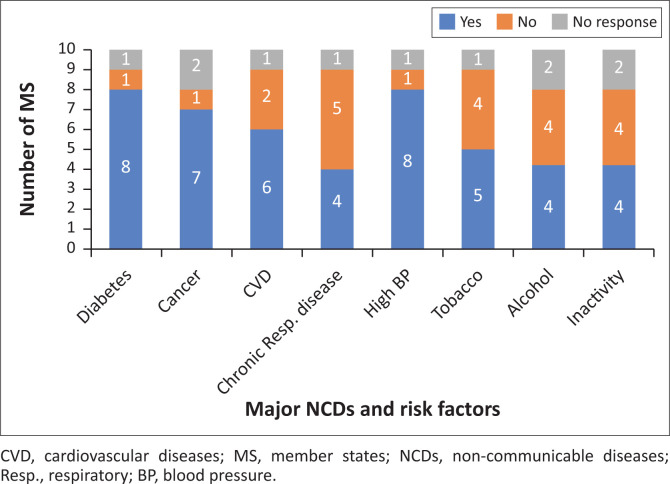
Number of member states reporting routine data collection on major non-communicable diseases and risk factors.

As Figure 1 shows, a majority of participating MS collect routine data on three major NCDs (diabetes > cancer > CVD) and on the risk factor of high blood pressure. Less than half routinely monitor chronic respiratory disease. About half of the participating MS routinely collect data on the risk factors: tobacco use, excessive alcohol consumption and physical inactivity. Four MS reported routine data on all four NCDs included in the survey, and three MS reported routine data on all four included risk factors.

**FIGURE 2:**
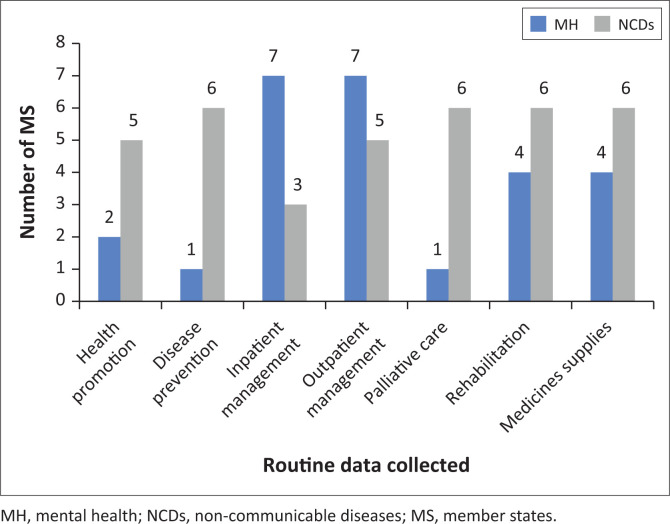
Number of member states reporting routine data collection on non-communicable diseases and mental health services and medicines.

Several MS collect routine data on NCDs and MH services and medicines. As Figure 2 depicts, most MS reported routine data collection on MH clinical management. The largest data gaps for routine MH services concern health promotion, disease prevention, rehabilitation, medicines and palliative care. Two-thirds of MS collect routine data on NCD prevention, medicines, palliative care and rehabilitation. The largest gaps in routine NCD service data existed for clinical management and health promotion.

## Challenges for routine non-communicable disease and mental health data collection during COVID-19 (from survey participants)

Commonly reported challenges to routine data collection on NCDs and MH included insufficient funds, inadequate human resources, lack of frameworks supporting data collection, lack of electronic data collection systems and limited integration of NCDs and their risk factors into existing surveillance systems. Some MS noted missing tools and indicator sets, others mentioned challenges in interoperability and creating enough priority for NCDs and MH to be included in health information systems (HIS) or surveillance. Further details on these major challenges and their key themes are provided in the following paragraphs, with selected verbatim answers included in-text (more in Box 1).

BOX 1 Member states responses on the question of challenges for routine data collection on non-communicable diseases and mental health.

**Challenges for routine NCD and MH data collection (from survey participants)**

1. **Insufficient funds:**
  - ‘There is a paucity of funds, and there is no funding partner’
  - ‘The five-year survey cycle is not respected due to a lack of financial resources’
  - ‘Financial and material resources are not available to conduct STEP WISE surveys on NCD risk factors’
2. **Inadequate human resource:**
  - ‘Not enough human resources; not enough financial resources for research’
  - ‘There is limited data collected on mental health at the level of the [*national indicators*] due to competition and limited space at the national level. There is also a need for a focal person to attend to data needs in the area of mental health and substance abuse who can also assist provincial focal persons for mental health to come up with responsive indicators to monitor the mental health programme at the [*provincial*] and [*district*] levels according to the individual provinces’ challenges’
  - ‘NCDs do not have dedicated data collectors or surveillance officers at facilities to capture data’
  - The five-year survey cycle is not respected due to a lack of qualified and available human resources (a great shortage)’.
3. **Lack of frameworks supporting data collection:**
  - ‘No national framework for doing this. No national mental health indicators’
  - ‘There are no means to collect this [*NCD*] data’
4. **Lack of electronic data collection systems:**
  - ‘Though data is collected at facility level, most data collection tools are paper-based, and electronic systems are not interoperable; hence, most data do not reach the national level. The NCDs programme lacks a good M&E system’.
  - ‘Insufficient funding to develop electronic systems for data collection. Competing priorities result in data on only certain conditions being included for collection’
  - ‘Limitations on inclusion of data elements and indicators in the only electronic system used to collect NCDs data in the country, the Web-based District Health Information System (DHIS)’
5. **Poor integration of NCDs and their risk factors into existing systems:**
  - ‘The existing indicators within the […] DHIS2 are not indicated’.
  - ‘Unwillingness of funders to include data collection for NCDs into their electronic platforms’.
  - ‘Insufficient synergy between programmes so as to address the dual diagnosed client’.

NCDS, non-communicable diseases; MH, mental health; M&E, monitoring and evaluation.

### Insufficient funds

Lack of adequate funding for NCD and MH data collection was a significant challenge stated by all MS in the survey. Participants alluded that this challenge persisted because little priority was given to NCDs and MH conditions despite their debilitating effects. Also, there are few donors offering funding for NCD or MH activities. Poor funding, including for staff and material, hinders many MS from conducting regular surveys.

### Inadequate human resources

Inadequate human resources for NCD and MH data collection was perceived by MS to be because of two major factors: insufficient funding and lack of qualified staff for data collection. For example, one participant mentioned that NCDs did not have dedicated data collectors or surveillance officers. Even though NCD and MH data collection require special training, data collectors for infectious diseases were mostly tasked in many countries to also collect NCD and MH data with very little or no prior training. The task of collecting NCD and MH data in addition to infectious disease data can be overwhelming for staff, and poor training can lead to low-quality reported data.

### Lack of frameworks supporting data collection

Frameworks provide a structured background, including protocols, which guide surveillance activities. These frameworks were found lacking which poses a challenge to standardised data collection and affects both the quality of data and the frequency of data collection. The absence of frameworks also suggests there is no formal system for costing NCD and MH activities.

### Lack of electronic data collection systems

Electronic databases provide a robust system for storing and sharing data. Paper-based data are difficult to store for a long time and to integrate into national health information systems. This also poses a threat to data safety. One participant explained that although data are collected at the facility level, most data collection tools are paper based and electronic systems are not interoperable. Member states stated that they were unable to use electronic systems because of financial resources to purchase electronic devices and software for data collection.

### Limited integration of non-communicable diseases and their risk factors into existing surveillance systems

Integrating NCDs and their risk factors into existing surveillance systems, such as for infectious diseases, is a recommended approach. It can improve surveillance for NCDs where infectious diseases have an established and robust mechanism for data collection. Unfortunately, many MS have not been able to achieve this integration. In some cases, this was because existing surveillance platforms were not ready or willing to integrate NCDs and MH. For example, one participant pointed out that some funders are reluctant to include NCD data in their electronic platforms.

### What are the perceived effects of COVID-19 on non-communicable diseases prevalence and incidence?

**FIGURE 3:**
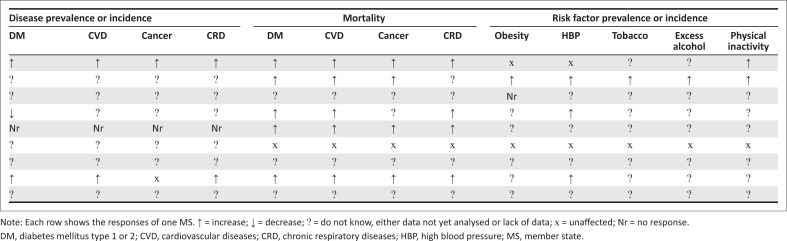
Perceived changes in non-communicable disease frequency, mortality and risk factors during COVID-19 pandemic.

Member states indicated a negative effect of the pandemic on NCDs, with an increase in NCD mortality, prevalence and risk factors (Figure 3). Increased mortality was reported by five out of nine MS for diabetes mellitus, CVD, cancer and chronic respiratory diseases. Two MS observed increases in NCD prevalence or incidence. The majority, however, did not have or had not yet analysed such data. Four MS reported increased NCD risk factor morbidity, mostly for high blood pressure and physical inactivity. As for disease data, the majority of MS also lack data on risk factors.

### How did the COVID-19 pandemic affect non-communicable disease services?

All MS indicated decreased access of NCD patients to healthcare services owing to COVID-19 measures, including a reallocation of healthcare staff and funding to the pandemic response. Three MS noted more interest and incentives for healthcare workers to work on COVID-19 response than on NCDs and five MS noted that funding for NCDs was diverted or decreased. The utilisation of services also decreased as patients feared contracting an infection at health facilities. Where specific data were available, several MS reported a decrease in NCD prevention (*n* = 5/9), outpatient care (*n* = 5/9), palliative care (*n* = 3/9) and rehabilitation. On the other hand, one or two MS noted an increase in each of these services; a majority (*n* = 5/9) indicated an increase in health promotion activities and one-third (*n* = 3/9) noted an increase in NCDs medicine supplies.

### Which data are available on the perceived effect of COVID-19 on mental health?

**FIGURE 4:**
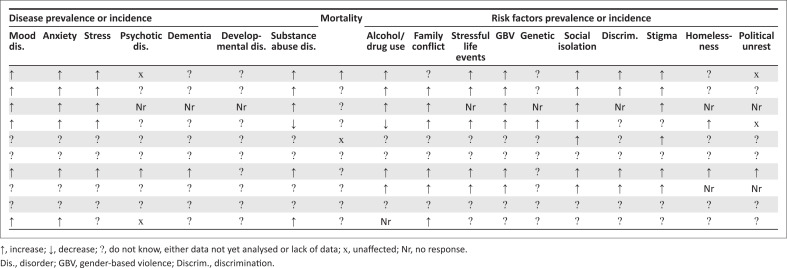
Reported effect of COVID-19 on mental health condition frequency, mortality and risk factors.

Survey responses included data on disease and risk factor incidence and prevalence as well as on MH-related mortality. A substantial negative impact of COVID-19 on MH was reported across countries (Figure 4). More than half of MS observed increases in mood, anxiety and stress disorders as well as substance abuse. A widespread lack of data or analyses on the impact of COVID-19 existed; however, particularly for dementia, psychotic disorders and developmental disorders. Eight out of 10 MS indicated an increase in at least one MH risk factor. Six out of 10 MS observed increases in substance abuse, gender-based violence (GBV), family conflict, social isolation and stigma.

### How did the COVID-19 pandemic affect mental health services?

Member states reported both MH service increases and decreases during the pandemic. Four out of the nine MS responding to this question indicated increases in MH promotion and medical supplies, which contrasted with a decrease in MH outpatient care services across MS. Causes for service disruptions were similar to those for NCDs.

### Are non-communicable diseases and mental health part of national health emergency preparedness and response?

Non-communicable diseases and MH were integrated into EPR plans in five out of 10 participating MS. One MS indicated that MH was integrated but not NCDs. Of the three MS without current integration of NCDs and MH, one MS indicated plans for integration.

### What action points have been and shall be developed?

The majority of MS (*n* = 7/9–10) implemented measures to minimise the effect of COVID-19 on NCDs and MH. Seven out of 10 MS took measures to overcome major MH service disruptions, including the integration of MH into essential healthcare (*n* = 6/10), telemedicine (*n* = 5/10), accredited platforms to offer health information, social protection measures (*n* = 4/10) and home healthcare (*n* = 3/10). Some MS expanded their MH capacity during the pandemic by recruiting more health workers and psychiatrists or establishing an MH commission and more psychiatric hospitals. Another example was the development and dissemination of guidelines for MH intervention during the pandemic, fostering awareness. Others ensured service availability for MH by allocating MH specialists to COVID-19 hospitals, using telemedicine and providing a round-the-clock emergency hotline. Six out of 10 MS reported no changes in MH data collection, while others noted additional ad hoc surveys or an increased frequency of data collection and expanded efforts to include vulnerable populations in data collection. Two MS suggested these improvements could be sustained. One MS noted that increased data collection ‘triggered the establishment of mental health psychosocial support teams’. During the course of the pandemic, three out of 10 countries collected data on symptoms or quality of life of patients following their severe acute respiratory syndrome coronavirus 2 (SARS-CoV-2) infection.

Six out of nine MS countered the disruption to NCD care with home healthcare (*n* = 5/6), integration of NCDs in essential healthcare (*n* = 4/6) and accredited platforms to offer health information, self-care and behaviour management (*n* = 3/6) or telemedicine (*n* = 2/6). While two MS reported a decrease in NCDs data collection during the pandemic, one MS reported ‘systematic NCDs and MH screening to identify undiagnosed cases since COVID-19 to was severe these patients [… *and* …] a priority for COVID-19 vaccination’. These changes were reportedly sustainable and resulted in policy changes for NCDs to enhance the identification of undiagnosed cases.

Strengthening health information and surveillance systems for NCDs and MH emerged as a key priority and opportunity. Building on surveillance advances during the COVID-19 pandemic, many MS expressed the need for better elaboration, integration, operationalisation and digitisation of NCDs and MH surveillance, and that this was a key area for Africa CDC support.

One MS stated that ‘MH data are collected at the level of the psychiatric structures during the annual supervisions. In 2022, with the parameterisation of the mental health module of the DHIS2 […], which has just been completed, we will be able to collect the data monthly. For the national survey, a mental health module has been integrated into the […] DHS survey since 2019. In the questionnaire for the facilities, drug and alcohol use are considered. In 2023, we will conduct a national mental health survey to further investigate these indicators’. Member states noted that African CDC could support their efforts, particularly through funding, technical assistance, training and prominent across responses, through strengthening surveillance systems.

## Discussion

Based on a survey, this study found that the COVID-19 pandemic was perceived to have negative effects on NCDs and MH in 10 AU MS. Member states observed increases in NCDs risk factors and mortality as well as elevated MH risk factors and morbidity during the pandemic. Many MS took steps to limit the negative impact of the pandemic on the NCDs and MH burden and the risk of widespread service disruptions; they also shared lessons to strengthen the integration of NCDs and MH into EPR for future pandemics. However, the study also identified that MS had varying NCDs and MH surveillance capacities because of key surveillance barriers. Because data and surveillance provide a critical public health foundation for preventing and controlling NCDs and MH routinely as well as during upcoming epidemics,^2^ these elements are discussed in particular.

Findings from this survey highlight how insufficient funding for NCDs and MH, which has been a key issue across many African countries,^18^ limits NCDI and MH surveillance in the participating MS. The absence of MH strategies in a majority of the selected MS also indicates a gap in MH governance. At the same time, the existence of NCDs or MH strategies in MS is not a guarantee for adequate resource allocation for their surveillance. There is a need for high political commitment and robust mechanisms that could track the proportion of resources allocated, distribution mechanisms of allocated resources and the impact of the resource allocation on NCDs and MH surveillance activities in the various MS.

Studies have shown that data gaps impede NCD and MH prevention and control systems.^2,19^ The resulting lack of MH promotion mechanisms and medicines in many MS negatively affects access to vital MH services and treatments. Even though some MS had routine data on MH clinical management, it remains unclear on which level, that is community, primary care level or specialised institutions. We also identified limited data on NCD clinical management in some MS, which is suggestive of limited attention to tracking the care cascade for chronic NCDs in these MS.

While almost all MS reported the availability of hospital data for NCDs and MH, several MS do not seem to use these for national surveillance activities. The low utilisation of the hospital data is likely because of the low quality of the data collected. It may also be because of other factors such as a lack of frameworks supporting data collection and data sharing, lack of electronic data collection systems and lack of human resources. For example, because most MS use paper-based systems for data collection, this could pose a barrier to sharing the data for decision-making.

Further, the poor integration of NCDs and MH indicators into existing surveillance systems in many MS signals a basic maturity level of NCDs and MH surveillance in these countries. Non-communicable diseases and MH surveillance systems in these MS could be strengthened through developing guidelines for surveillance and core indicators and by engaging MS to share experiences and co-create surveillance mechanisms based on their country’s needs, strengths, weaknesses, opportunities and threats.

A promising development we identified is the inclusion of selected NCDs and MH conditions in the third edition of the Integrated Disease Surveillance and Response Strategy (IDSR) guidelines. However, we could not ascertain the level of implementation of integrated surveillance across NCDs, MH and infectious diseases. In practice, the operationalisation of indicators and integration with existing infectious disease surveillance may be challenging, especially in light of the inadequate human resources for surveillance mentioned by many MS. A successful integration of NCD and MH surveillance into existing surveillance systems, including platforms of donor-funded infectious disease programmes, requires overcoming currently still existing political and administrative resistance.

In addition, programmes to strengthen the healthcare workforce for future public health emergency preparedness and response need to include planning for skilled routine as well as surge capacities to ensure the continuity of prevention and care for NCDs and MH.

Strengthening NCDs and MH surveillance systems and integrated EPR across the African region could benefit from closing remaining evidence gaps, including (1) NCDs components in EPR plans and how these have been implemented during the pandemic, (2) long-term effects of the pandemic on the NCDs and MH burden across the continent and regarding NCD and MH surveillance, (3) governance, including policies and administrative models, (4) assessments of the national surveillance system capacities and data flows, (5) innovative data collection and analysis approaches, (6) costs, funding and budgeting practices for surveillance activities, (7) human resource needs and capacities and (8) integration into existing systems.

### Limitations

The survey for this study was a pilot exercise whose findings are not representative of the continent. It attempted to provide a balanced sample of franco- and anglophone MS with two countries from each AU region. We asked respondents to indicate whether the prevalence and incidence of major NCDs and MH conditions and their risk factors had increased, decreased or remained unchanged during the COVID-19 pandemic; we did not, however, require that the respondents specify disease and risk factor frequencies in numbers or data sources. Further, the survey may have benefited from input from surveillance and EPR focal points; however, NCDs and MH focal points were encouraged to seek support from national thematic experts if additional expertise was required to respond to questions. This measure was to reduce response and non-response bias, which may have affected the results. The strong response and completion rate suggest a low level of non-response bias to the findings. The findings of this exploratory survey provide a basis for further data collection and analyses regarding the effects of COVID-19 on these variables.

## Conclusion

The 10 surveyed countries on the African continent had varying levels of national NCD and MH surveillance capacities and practices. Expanding NCD and MH surveillance in these MS, as part of national strategies to strengthen the health information system, requires adequate funding, sufficient human resources and political support. Eventually, robust NCD and MH data from such surveillance will close the data gaps observed in this survey and provide evidence to decision-makers for the establishment of targeted and cost-effective NCD and MH programmes and interventions.

Where data were available, countries mostly registered negative effects of the COVID-19 pandemic on major NCDs and MH conditions, several of their risk factors and interruptions in the continuity of key health services. Despite gaps in emergency and response plans and systems that integrated NCDs and MH, many countries implemented innovative countermeasures towards ensuring continuity of care. Countries may benefit if they strengthen NCD and MH policies and surveillance and, at the same time, integrate NCDs and MH further into national emergency preparedness and response (EPR) planning.

Public health emergencies, such as the COVID-19 pandemic, further highlight the need for integrated surveillance and response. As a continental public agency, the Africa CDC can guide and facilitate MS collaboration aimed at closing NCD and MH data gaps and decreasing continental vulnerability to the effects of public health emergencies on NCDs and MH.
